# Evaluation of the Antiviral Activity against Infectious Pancreatic Necrosis Virus (IPNV) of a Copper (I) Homoleptic Complex with a Coumarin as Ligand

**DOI:** 10.3390/molecules27010032

**Published:** 2021-12-22

**Authors:** Daniela Gutiérrez, Almendra Benavides, Beatriz Valenzuela, Carolina Mascayano, Maialen Aldabaldetrecu, Angel Olguín, Juan Guerrero, Brenda Modak

**Affiliations:** 1 Laboratory of Natural Product Chemistry, Center of Aquatic Biotechnology, Department of Environmental Sciences, Faculty of Chemistry and Biology, University of Santiago de Chile, Santiago 3363, Chile; daniela.gutierrez@usach.cl (D.G.); almendra.benavides@usach.cl (A.B.); beatriz.valenzuelam@usach.cl (B.V.); angel.olguin@usach.cl (A.O.); 2 Laboratory of Computational Simulations and Rational Drug Design, Department of Environmental Sciences, Faculty of Chemistry and Biology, University of Santiago de Chile, Santiago 3363, Chile; carolina.mascayano@usach.cl; 3 Laboratory of Coordination Compounds and Supramolecularity, Faculty of Chemistry and Biology, University of Santiago of Chile, Santiago 9170002, Chile; maialen.aldabaldetrecu@usach.cl

**Keywords:** coumarin, copper (I), coordination compounds, IPNV, antiviral activity

## Abstract

The aquatic infectious pancreatic necrosis virus (IPNV) causes a severe disease in farmed salmonid fish that generates great economic losses in the aquaculture industry. In the search for new tools to control the disease, in this paper we show the results obtained from the evaluation of the antiviral effect of [Cu(NN_1_)_2_](ClO_4_) Cu(I) complex, synthesized in our laboratory, where the NN_1_ ligand is a synthetic derivate of the natural compound coumarin. This complex demonstrated antiviral activity against IPNV at 5.0 and 15.0 µg/mL causing a decrease viral load 99.0% and 99.5%, respectively. The Molecular Docking studies carried out showed that the copper complex would interact with the VP2 protein, specifically in the S domain, altering the process of entry of the virus into the host cell.

## 1. Introduction

Aquaculture is an important source of food, nutrition, income, and livelihoods for hundreds of millions of people around the world. However, with intensification of the production, viral diseases have emerged representing a challenge to sustainable development. The aquatic infectious pancreatic necrosis virus (IPNV) causes infectious pancreatic necrosis (IPN), a severe disease in farmed salmonid fish that causes great economic losses in the aquaculture industry. At present, IPNV is among the most persistent salmonid pathogens in Chile [1]. This virus belongs to the genus Aquabirnaviru family *Birnaviridae* [2]. During an IPN outbreak, mortality rates can vary greatly from insignificant to almost 100%, and these differences have been ascribed to several environmental, viral and host-related factors [3]. The survivors of an infection become asymptomatic carriers of the virus, even for years, acting as reservoirs of the virus and spreading it through the water via feces, and mainly during stress episodes, but essentially as breeders through their reproductive products [4]. In addition to the mortality caused directly by viral infection, the virus also causes immunosuppression in fish, making them more vulnerable to other pathogens [5].

The genome consists of two double-stranded RNA segments, packed in a non-enveloped single-shelled icosahedrical capsid [6]. Segment A codes for three viral proteins: two of the structural type, VP2 and VP3, and one non-structural VP4 [7]. VP2 is processed during virus maturation [8]; it is the main component of the outer capsid of the virus and is directly associated with the production of specific neutralizing antibodies against the virus [9], this being one of the main virulence determinants in IPN. The VP3 protein is part of the internal capsid of the virus, which binds to RNA, forming a ribonucleoprotein in the central structure of the virus [10]. Segment A also encodes a non-structural arginine-rich protein called VP5. This protein has been detected in infected cells. However, it has recently been shown that this protein is dispensable for in vivo viral replication and is not involved in persistent infection or virulence of the virus, but it could have antiapoptotic activity [11,12]. Segment B codes for a protein called VP1, which is associated with the replication of the viral genome [13,14].

Due to the significant economic impact of the disease in the aquaculture industry, a great effort has been made to control IPN by different methods, mainly through vaccine design. At present, several vaccines are available against IPNV. They are either subunit vaccines or inactivated products. The subunit vaccines contain as a major viral antigen VP2 expressed in *Escherichia coli*, which seeks to stimulate immune response by producing neutralizing antibodies. However, it is known that these vaccines have not been sufficiently protective against IPNV infection [15]. Similarly, neither the viral-like particle (VLP) vaccine, which resembles a viral capsid, nor DNA vaccines, which provoke viral protein expression within the cell, elicit protective immunity against IPNV [5]. In addition, IPNV is a very tough virus, is extremely resistant to most disinfectants [16], survives in both fresh water and seawater for considerable periods [17], and is resistant to high temperature [18]. Therefore, there is a need to seek new tools for its control.

On the other hand, the crucial role of copper and its derivatives as important bio-active compounds have led to much interest in them as potential drugs for treatment of several diseases. There is extensive information on its bioinorganic properties and its mode of action in various biological systems as antimicrobial, antiviral, an-ti-inflammatory, antitumor, and enzyme inhibitors compounds [19]. With that in mind, we have synthesized a complex [Cu(NN_1_)_2_]ClO_4_, where the NN_1_ ligand is a synthetic derivate of the natural compound coumarin (NN_1_ = 6-((quinolin-2-ylmethylene)amino)-2H-chromen-2-one) which showed remarkable antibacterial activity against *F. psychrophilum*, a pathogen that attacks salmon [20] and in marine bacterium *V. harveyi* [21]. In the previous paper [20] was indicated that we used the coumarin (1-benzopyran-2-one) as one of the ligand fragments since its known biological properties [22], while the quinoline fragment was used of its ability to stability of the Cu(I) ion.

Based on this background, in this work, we evaluate the antiviral activity of this copper complex against IPNV and propose that activity would be explained by interactions between protein target of the virus and the metal complex based on molecular docking simulations.

## 2. Results and Discussion

### 2.1. Chemical Compunds

**Figure 1:**
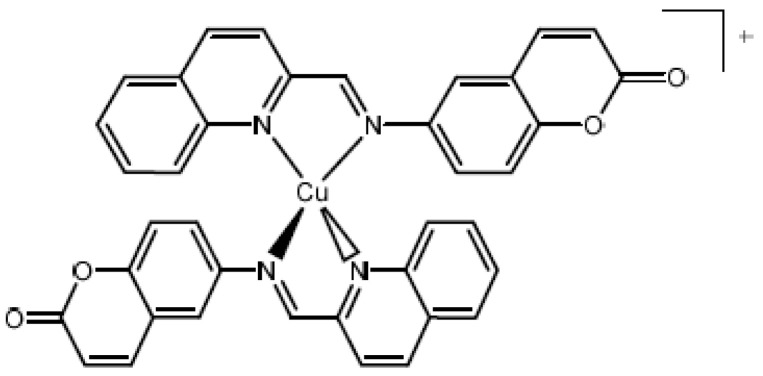
Chemistry Structure of Copper (I) Complex [Cu(NN_1_)_2_]ClO_4_. Where NN_1_ is 6-((quinolin-2-ylmethylene)amine)-2H-chromen-2-one.

Copper (I) complex, [Cu(NN_1_)_2_]ClO_4_ (Figure 1), previously synthesized and characterized by NMR techniques, UV–Vis, and cyclic voltammetry [20], was used to test the antiviral effect on IPNV. Coumarin and copper (I) salt [Cu(CH_3_CN)_4_]ClO_4_, precursors of the synthesis of the copper (I) complex, were used as compound effect controls.

### 2.2. Cytotoxicity Test

**Figure 2:**
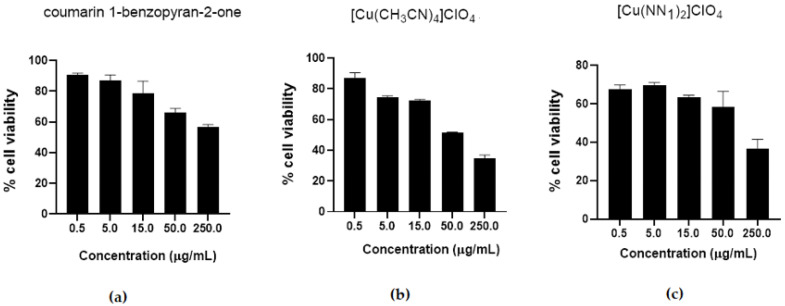
Determination of cytotoxicity of test compounds on CHSE-214 cells. CHSE-214 cells were treated with 0.5, 15.0, 50.0 and 250.0 µg/mL of (**a**) coumarin, (**b**) ([Cu(CH_3_CN)_4_]ClO_4_) and (**c**) copper(I) complex [Cu(NN_1_)_2_]ClO_4_ and viability was determined by flow cytometry.

In order to determine the working concentrations of test compounds, first the cytotoxicity of the copper (I) complex [Cu(NN_1_)_2_]ClO_4_ and its precursors, coumarin and [Cu(CH_3_CN)_4_]ClO_4_ was evaluated. Viability of CHSE-214 cells (Chinook salmon embryo CHSE-214, ATCC CRL 1681) was determined after treatment with the different compounds in a range of concentrations between 0.5 and 250 µg/mL, incubated at 16 °C for 24 h by flow cytometry and the results are shown in Figure 2. In the coumarin and [Cu(CH_3_CN)_4_]ClO_4_, both precursors of the Cu(I) complex, a concentration-dependent toxicity was observed, while the copper(I) complex maintains a relatively constant cell viability between 0.5 and 50 µg/mL, although it is still the one with the highest toxicity. On the other hand, in all compounds, concentrations higher than 15 µg/mL, decreased cell viability by up to 60% in CHSE-214 cells. The results show a similar cell viability for both copper compounds above 50%, however, the focus of this work is to obtain a drug, ensuring the stability of copper complex (I) and with a lower amount of copper per mass of compound administered. According to these cell viability assays, a concentration less than 15 µg/mL was used for subsequent tests.

### 2.3. Antiviral Activity

**Figure 3:**
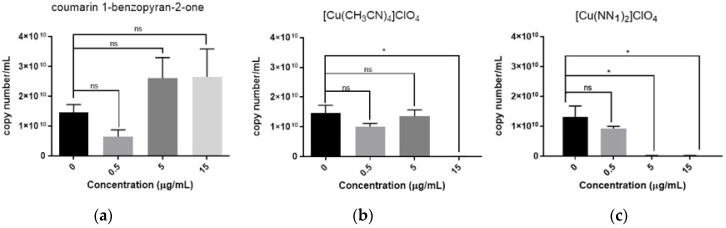
Antiviral activity against IPNV of test compounds in CHSE-214 cells. IPNV was incubated for 24 h with 0.5, 5.0, and 15.0 µg/mL of (**a**) coumarin, (**b**) [Cu(CH_3_CN)_4_]ClO_4_, (**c**) [Cu(NN_1_)_2_]ClO_4_ complex, after that CHSE-214 cells were treated for 24 h with IPNV. RNA total extraction was performed from the cell cultures and viral load was determined by quantitative real-time PCR with three technical replicates. Statistical differences were determined by one-way ANOVA test (* *p* < 0.05; ns = non-significant).

Viral replication was tested in order to evaluate the antiviral activity of coumarin, the [Cu(CH_3_CN)_4_]ClO_4_ precursors and [Cu(NN_1_)_2_](ClO_4_) complex, on CHSE-214 infected cells. The cells were inoculated with the compounds pre-treated with virus and the viral load at 24 h post infection was measured. The total abundance of viral transcripts was quantified by RT-qPCR. The results showed that cultures infected with viruses previously treated with 5.0 and 15.0 µg/mL of coumarin present a high viral load (Figure 3a). In contrast, the treatment with [Cu(CH_3_CN)_4_]ClO_4_ non decrease significantly viral load at 0.5 and 5.0 µg/mL in the cells (Figure 3b), but at 15.0 µg/mL we observed that this compound almost entirely reduces viral load in CHSE 214 cells (99.5%) relative to cells infected without treatment. While, the cells infected treated with [Cu(NN_1_)_2_](ClO_4_) complex at 5.0 and 15.0 µg/mL showed a decrease viral load 99% and 99.5% respectively (Figure 3c). Although the precursor [Cu(CH_3_CN)_4_]ClO_4_) complex showed high activity at 15 µg/mL, similar to that of the complex [Cu(NN_1_)_2_](ClO_4_), it is important to consider that the complex has 2.5 times less copper than an equimolar amount of precursor complex. This is relevant at the time of to consider the toxicity of this metal at high concentrations. For example, it is known that copper in high quantities can alters swimming fish; causes oxidative damage; disrupts osmoregulation structure and pathology of kidneys, liver, gills, and other stem cells; impacts mechanoreceptors of lateral line canals; and impairs functions of the olfactory organs and brain, corticosteroid metabolism, and gene transcription and expression, among other factors [23].

Although it is not possible to mean synergism since coumarin alone has no activity, it is clear that the presence of this natural compound is necessary to improve the antiviral capacity of the copper complex in relation to the precursor complex. Previous studies performed with [Cu(NN_1_)_2_]ClO_4_ complex shown that this complexes also exhibited a improved antibacterial activities relative to coumarin and [Cu(CH_3_CN)_4_]ClO_4_ precursors. Those results were explained by an increase of the lipophilicity of the complexes, which occurs after the complexation of the organic residue to copper ion, which favors their transfer across the lipid membrane of the bacterial cell wall [24] in accordance with Tweedy’s chelation theory. So, the formed complex has a more lipophilic character compared to precursors alone and therefore can more easily penetrate the lipid layers of the bacterial cell membrane [25].

In view of the observed activity of copper (I) complex [Cu(NN_1_)_2_]ClO_4_ against IPNV, coumarin moiety would be exerting an effect in the complex-virus interaction. The lipids of the viral envelope form a continuous bilayer that functions as a permeability barrier protecting the viral capsid from the external medium. Embedded in this bilayer are numerous copies of a limited number of virally encoded transmembrane proteins that are required for virus entry into a potential host cell. These proteins mediate two essential functions: attachment of the virus to the cell surface, and fusion of the viral envelope with a cell membrane (resulting in accession of the viral capsid containing the genome to the cellular cytoplasm) [26].

An antiviral compound can inhibit viral infection through three approaches: (a) direct contact and destroying virus; (b) preventing adsorption and penetration viral into the cells; and (c) impeding replication, assembly, and maturation of viral particle [27]. Since VP2 is the main viral capsid protein, which is targeted by type-specific neutralizing antibodies, and also acts as the cell-binding protein [4]. So, we hypothesize that the antiviral activity of complex can be associated with an interaction of complexes with the membrane proteins virus, inhibiting their capacity for absorption in the cellular membrane.

### 2.4. Computational Studies

In order to obtain information about the mechanism of the antiviral action, we evaluated the affinity between [Cu(NN_1_)_2_]ClO_4_ complex and VP2 protein through molecular modeling (MD).

To corroborate the adequate structure of Cu(I) complex before be used in molecular docking simulations, the structural geometry of free [Cu(NN_1_)_2_]ClO_4_ and its UV-Vis spectrum were simulated. 

The simulated spectrum (Figure S1) shows a broad band with three contributions corresponding to the expected MLCT for a characteristic copper (I) homoleptic complex that is very close to the profile of the experimental spectrum showing good agreement between both the optimized and the experimental structure [20]. Considering the high dependence of the properties of copper (I) complexes on the geometry conditioned by the ligands around the center of the metal, the observed spectral similarity is a good starting point for the following molecular docking calculations.

**Figure 4:**
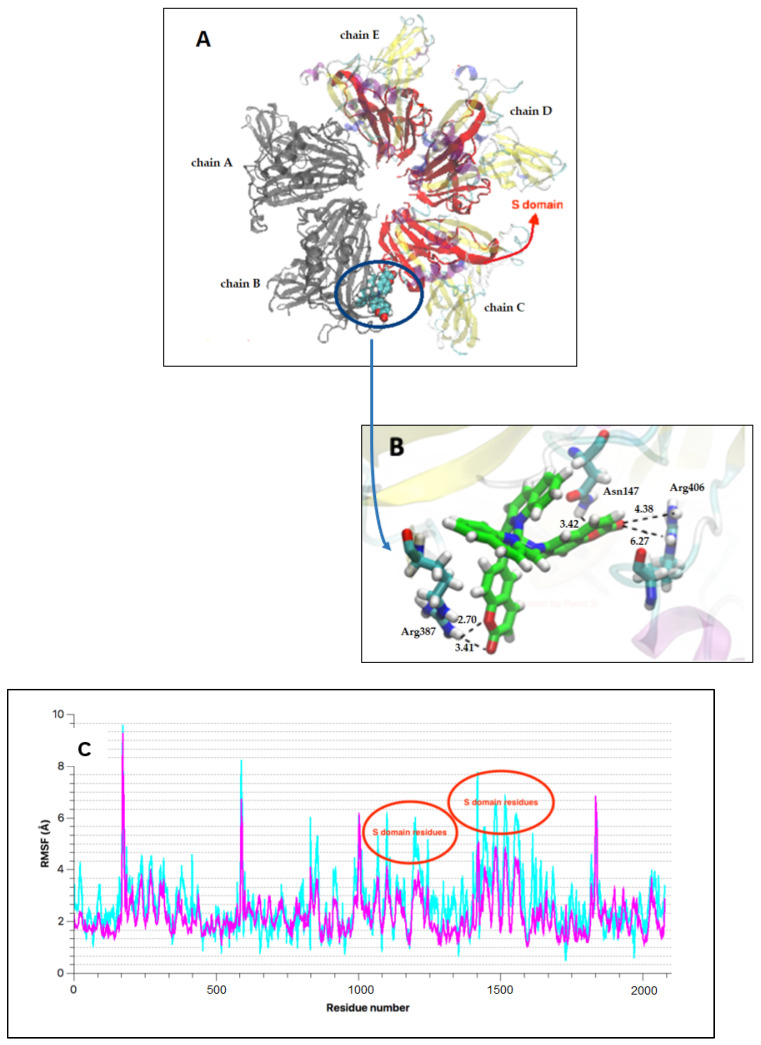
(**A**) 3D representation of the chains (colored) and S domain (red) affected by ligand binding (colored); (**B**) binding interaction between complex and protein; (**C**) RMSF profile between initial state (fuchsia) and final state (blue light) and their corresponding residues.

The MD showed the stabilization of the VP2-complex assemble during a short simulation of 16 ns. We observed that the Cu(I) complex was located into the interphase of VP2 (Figure 4A) generating short H…O noncovalent interaction and H-bonds between the coumarin fragments of NN_1_ ligand and the residues of the protein as Arg (387 and 406) and Asn 147, with distances in the range 2.4 to 4.2 Å (Figure 4B). Additionally, we obtained the root-mean-square fluctuation (RMSF) of protein binding with the ligand along to all MD. The graphical showed that the most fragment perturbed was the S domain (Figure 4A,C) by effect of the ligand-binding and VP2 (Eb −78.49 Kcal/mol). VP2 is constituted of three domains: a central (called S) which in subviral particles constitutes the shell, playing a key role during virus entry into the cell and being responsible for receptor recognition; the base (called B), which is in the inner side of the particle; and the spike or projection (called *p*) to the outside of the capside [4]. Thus, the copper complex would interact with the VP2 protein, specifically disturbing the S domain, altering the process of entry of the virus into the host cell, what is reflected in the antiviral activity that we determine in the previous experiment.

Additionally, several studies using nucleotide sequence analyses have confirmed that the VP2 residues 217 and 221 are the major determinant of virulence of IPNV serotype Sp strains [28]. It has described that highly virulent isolates possess residues Thr217 and Ala221; moderate to low-virulence strains have Pro217 and Ala221, while the strains containing Thr221 are almost avirulent. On the other hand, in CHSE-214 cells, it has been calculated about 6000 receptors available for VP2-IPNV fixation [29], however, only a part of them is being used by the virus [4]. Our results showed that the main interactions observed were with the residues Arg 387 and 406 and Asn 147. Therefore, the copper complex would not be binding to the VP2 residues described as the most important in determining virulence, but indicated that Cu(I) complex interact with a region of dominium S.

Consequently, we suggest that the copper (I) complex would be inhibiting the entry of the virus blocking sites involved with the fixation and internalization of virus to the cell. However, it is important to know the invasion mechanism of IPNV completely to understand the antiviral effect of complex.

## 3. Materials and Methods

### 3.1. Cytotoxicity Test

Monolayers of CHSE-214 cells were cultured at 16 °C in Minimum Essential Medium (MEM) (Corning) supplemented with 10% fetal bovine serum (FBS) (Hyclone, Thermo Scientific Logan Utah, EE. UU), 4 mM *L*-glutamine (Gibco), 40 μM 2-mercapthoetano, non-essential amino acids (Gibco), Hepes (Gibco) and 50 μg/mL gentamicin. To discard toxicity of test compounds, 1 × 10^5^ CHSE-214 cells were treated with different doses of coumarin, Cu(I) precursor complex and [Cu(NN_1_)_2_]ClO_4_ complex in a range of concentrations between 0.5 μg/mL to 250 μg/mL solubilized in dimethylsulfoxide (DMSO, Merck, Darmstadt, Germany) and incubated in supplemented MEM (Corning) for 24 h at 16 °C. Later, the cells were collected, pelleted and resuspended in 300 μL of IF (Phosphate Buffered Saline and 2% fetal bovine serum) and 2 μL of propidium iodide (PI, 1 mg/mL) was added. Viable cells (PI negative cells) were quantified by flow cytometer using FACSCanto II Cytometer (BD Biosciences, University of Santiago of Chile, Santiago, RM, Chile). As positive control, cells were incubated without compounds; for death control, cells were incubated with 30% ethanol; medium control cells were treated with medium containing 1% of DMSO.

### 3.2. Antiviral Activity Evaluation

#### 3.2.1. IPNV Propagation

IPNV sp strain was propagated in monolayers of the Chinook salmon embryo CHSE-214 in minimal Eagle’s medium (MEM) supplemented with 2% bovine fetal serum (BFS), 6 mM *L*-glutamine, 40 µM 2-mercaptoethanol and 50 µg/mL gentamicin at 16 °C. The cells were incubated until cytopathic effect greater than 90% was observed. Virus titer was determined by plaque assay lysis [30].

#### 3.2.2. Antiviral Activity and Viral Load Assay

CHSE-214 cells were grown at 90% confluence in 24-well plates. The cells were infected with IPNV at a multiplicity of infection (MOI) 0.1 pre-treated with 0.5, 5 and 15 µg/mL of each test compound for 24 h and cells without compounds. The cultures infection was performed to 24 h at 16 °C. A group of cells was incubated with vehicle control (0.02% DMSO). After incubation, the cells were collected and dissolved in 1 mL of TRIsure (Bioline, London, UK) and stored at −20 °C until further extraction of total RNA.

#### 3.2.3. RNA Extraction and cDNA Synthesis

Total RNA was extracted according the manufacturer’s protocol and then dissolved in pyrogen-free DEPC-treated water (Invitrogen) and stored at −80 °C. In all cases, RNA was quantified by UV spectrophotometry (Nanoquant Infinite M200 pro (TECAN, Austria) and analyzed by electrophoresis on agarose gels to verify integrity. RNA (2 µg) was treated with RQ1 RNase free DNase (Promega Corporation, Madison, WI, USA) and cDNA synthesis was carried out using reverse transcriptase M-MLV (Promega) and Oligo dT (Promega) and the manufacturer’s protocol.

#### 3.2.4. Real Time Quantitative PCR

The viral load was determined by real time quantitative reverse transcription-PCR (real-time qRT-PCR). Real Time quantitative PCR reactions were carried out in 96-well reaction plates (PCR Microplate, Axygen, Sigma Aldrich, St. Louis, MO, USA) using SensiMixTM SYBR HI-ROX mastermix (Bioline) in Stratagene Mx3000p equipment (Agilent Technologies, Waldbronn, Germany). Reaction mixtures were performed in triplicate, qRT-PCR was performed with a SensiMix SYBR HI-ROX with 2X SYBR Green PCR Master Mix (Bioline) following the manufacturer’s protocol and cDNA. The PCR primer sequences are listed in Table 1. The cycling conditions were: 95 °C for 5 min, followed by 40 cycles consisting of 95 °C for 15 s, 60 °C for 15 s, 72 °C for 30 s. PCR product quality was monitored using post-PCR melt curve analysis. Data were analyzed using MxPro QPCR software (Agilent Technologies, USA). The expression was normalized against elongation factor 1α (ef1a) and presented as 2−ΔCT.

### 3.3. Computational Studies

The structure was built with the GaussianView5 software [31]. The ChelpG charges were obtained at the B3LYP/LanL2dz theory level employing the Gaussian 09 package [32]. Docking studies of the inhibitor and the crystal structures of polymerase from infectious pancreatic necrosis virus (IPNV) (PDB code: 3IDE, 3.35 Å resolution) [33] were performed with the AutoDock4 package [34] using a Lamarckian algorithm and assuming total flexibility of the inhibitors. The multiple grid maps were made up of 126 × 126 × 126 for protein, with a grid-point spacing of 0.375 Å. The AutoTors option was used to define the ligand torsions, and the docking results were then analyzed by a ranked cluster analysis, resulting in conformations and best position with the highest overall binding energy (most negative −ΔG binding value).

Molecular docking (MD) simulations were performed using the NAMD 2.13 software [35] using the Charmm27 force field. The complex between the molecule and IPNV was placed in a 100 × 200 × 200 Å water box (116,440 waters) and neutralized with NaCl, with a cutoff of 10 Å of nonbonding interactions [36]. Periodic boundary conditions were applied with constant volume and pressure. A general protocol was followed, employing a cutoff value of 10 Å, and comprising an initial phase of 50,000 steps of minimization, followed by 50 ps of heating from 0 K up 288 K by the Langevin method and finally 16 ns of simulation were performed.

The B3LYP/LANL2DZ in Gaussian 09 package was performed to simulate the UV spectrum used to validate the ligands conformation around metal center and correlated with the experimental spectrum of complex. 

### 3.4. Statistical Analysis

Statistical analyses of gene expression were performed using the GraphPad Prism 8.0 software. Statistical differences were determined by one-way ANOVA test (* *p* < 0.05; ** *p* < 0.01; *** *p* < 0.001). Data are representative of three independent experiments.

## 4. Conclusions

An in vitro study carried out against IPNV showed that [Cu(NN_1_)_2_]ClO_4_ complex increased its antiviral capacity compared to precursor ([Cu(CH_3_CN)_4_]ClO_4_) complex to tested concentrations, while coumarin showed no activity. However, it is clear that the inclusion of this natural moiety into NN_1_ ligand is necessary to improve the antiviral ability of the copper complex in relation to the precursors.

The results obtained with MD showed that the copper (I) complex was located between the interphases of VP2 protein forming short H…O noncovalent interaction with the coumarin fragments of NN_1_ ligands. These interphases corresponding to the shell of the capsid, being able to affect the entry/attachment of the virus into the cell, which would explain the antiviral activity observed in vitro. Thus, this compound emerges as a promising candidate for further evaluation in an in vivo model.

## Figures and Tables

**Table 1:** PCR primers sequences used in gene expression studies (forward and reverse primer).

Target	Primer Sequence (5′-3′)	Accession Number
VP2	ACCAAGTTCGACTTCCAGC ATCGGCTTGGTGATGTTCTC	GenBank: FN257531.1

## Data Availability

Not applicable.

## Supplementary Materials

The following supporting information can be downloaded. Figure S1: UV-Vis spectrum simulation of [Cu(NN1)_2_]ClO_4_ complex.

## Sample Availability

Samples of the [Cu(NN_1_)_2_]ClO_4_ complex are available from the authors.
