# Supplementary figures and images for: Evaluation of the Antiviral Activity against Infectious Pancreatic Necrosis Virus (IPNV) of a Copper (I) Homoleptic Complex with a Coumarin as Ligand
Source: Molecules. 2021 Dec 22;27(1):32. doi: 10.3390/molecules27010032 (PMC8746282; doi:10.3390/molecules27010032)

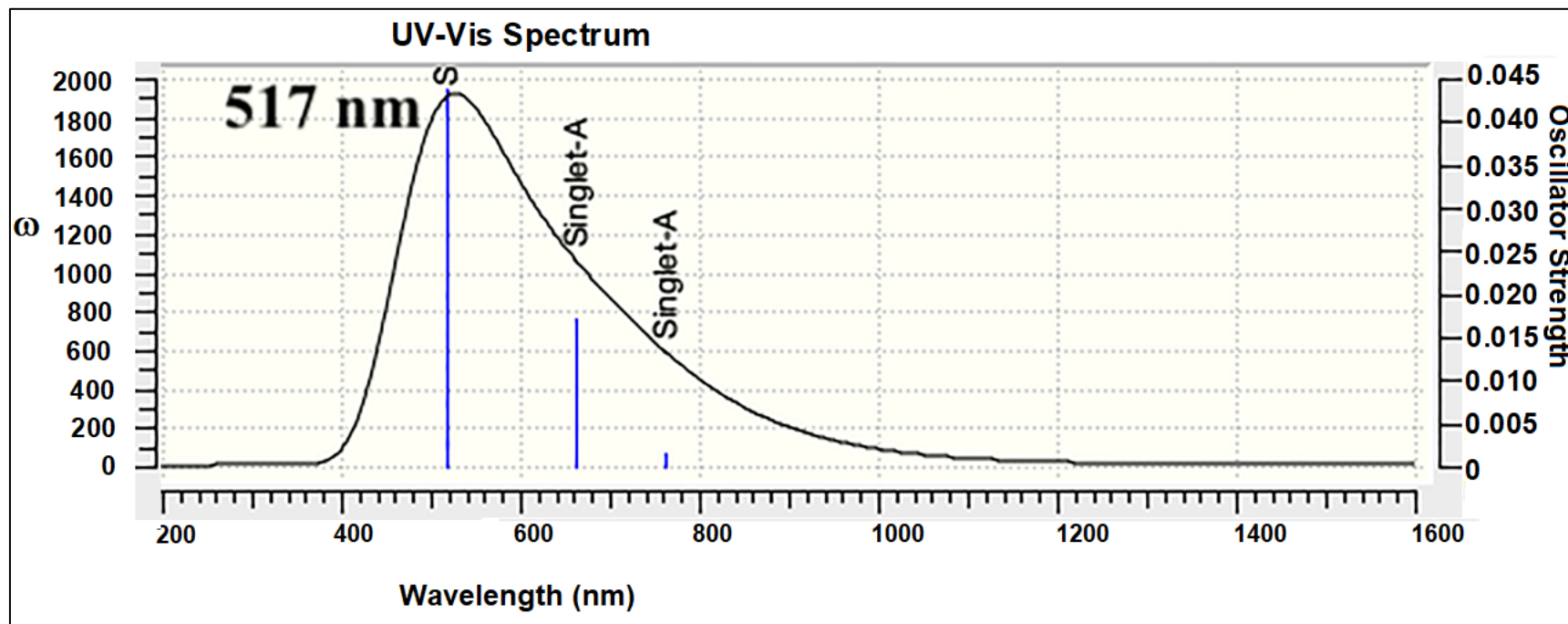

Figure S1: UV-Vis spectrum simulation of [Cu(NN1)<sub>2</sub>]ClO<sub>4</sub> complex.

Supplement: Supplementary file 1 [file molecules-27-00032-s001.zip › molecules-1505287-supplementary.pdf]
